# Gender differences measured by the MATRICS consensus cognitive battery in chronic schizophrenia patients

**DOI:** 10.1038/s41598-017-12027-w

**Published:** 2017-09-19

**Authors:** Baohua Zhang, Mei Han, Shuping Tan, Fu De Yang, Yunlong Tan, Shurong Jiang, Xiangyang Zhang, Xu-Feng Huang

**Affiliations:** 1 Beijing HuiLongGuan Hospital, Peking University, Beijing, PR China; 2 School of Medicine, University of Wollongong, and Illawarra Health and Medical Research Institute (IHMRI), Wollongong NSW 2522, Australia; 3 Department of Psychiatry and Behavioral Sciences, The University of Texas Health Science Center at Houston, Houston, TX USA

## Abstract

Using Repeatable Battery for the Assessment of Neuropsychological Status (RBANS), previous study showed significant gender differences for cognitive deficits in immediate and delayed memory in schizophrenia patients. However, RBANS does not include reasoning and problem solving, and social cognition. These cognitive functions can significantly affect the outcomes and daily life in patients. This study examined the gender differences of cognition using the measurement and treatment research to improve cognition in schizophrenia (MATRICS) consensus cognitive battery (MCCB), especially focusing on reasoning and problem solving, and social cognition in schizophrenia patients. The results showed that healthy controls exemplified better cognition than patients in both genders in all examined MCCB scores. Male healthy controls had better reasoning and problem solving and working memory than females, but these gender differences were not presented in schizophrenia patients. Also, male schizophrenia patients showed worse cognition than females on social cognition, processing speed, verbal learning and visual learning. Our results support that male schizophrenia patients had more cognitive impairment than females on reasoning and problem solving, social cognition, processing speed, working memory, verbal learning and visual learning.

## Introduction

Cognitive deficit is one of the core features of schizophrenia^1^. Cognitive deficit directly affect the quality of life and employment in schizophrenia patients^2^. Gender differences are recognized in brain neurotransmitter and receptors and function as well as their influence in behavior including in schizophrenia patients. Schizophrenia patients show gender differences in clinical symptoms, course and treatment outcome^3^. Several studies have reported gender differences in relation to cognitive deficits in schizophrenia^4–6^. An in-depth investigation of gender difference of cognitive deficits could help us to guide preclinical and clinical research and possible new ideas for a better treatment for patients.

Although we and others have used the repeatable battery for the assessment of neuropsychological status (RBANS) to report that the immediate memory and delayed memory were poorer in male than female chronic schizophrenia patients^7–9^. RBANS was initially introduced to provide neuropsychological assessment in screening for dementia^10^. It has since been shown to be valid for other mental illnesses including schizophrenia^11^. The major weakness of RBANS is that its cognitive measurement does not include reasoning and problem solving, and social cognition. These cognitive domains can significantly affect quality of life and independent living skills in schizophrenia, including relapse, worsened symptoms, and resulting unemployment^12–15^. The gender differences of cognitive impairment on reasoning and problem solving, or social cognition have not been systemically investigated in schizophrenia patients. In addition, some studies failed to find any gender differences of cognitive deficits in schizophrenia patients, or found better cognition in male schizophrenia patients than females^16,17^. Therefore, the gender differences of cognitive function in schizophrenia patients remained controversial and deserve further investigation.

Measurement and treatment research to improve cognition in schizophrenia (MATRICS) was originally developed by the US National Institute of Mental Health (NIMH). The primary goal of the NIMH-MATRICS was to encourage the development of pharmacological agents to improve cognition in schizophrenia^18^. The MATRICS consensus cognitive battery (MCCB) was widely developed to measure cognitive function in schizophrenia patients after the cognitive measure method has been evaluated based on its test-retest reliability, high utility as a repeated measure, relationship to functional outcome, practicality and tolerability, which was accepted by most medical researchers^18^. Importantly, the MCCB cognitive measure includes reasoning and problem solving and social cognition, which were important domains of cognitive deficits in schizophrenia. In recent years, MCCB for cognitive measurement has been translated into Chinese, and its clinical validity and test-retest reliability have been established between schizophrenia patients and health controls^19^. The MCCB has been applied in cognition studies for schizophrenia patients in Chinese population^20,21^.

To the best of our knowledge, no previous study has investigated the gender differences of cognitive deficits in schizophrenia patients in the Chinese Han population using MCCB. The purpose of this study was to verify: (1) whether or not schizophrenia patients had gender differences in cognitive deficits for reasoning and problem solving, and social cognition; (2) if the gender differences identified in our previous study^9^ using RBANS can be confirm using MCCB; and (3) whether the gender differences of cognitive deficits in schizophrenia were associated with specific clinical characteristics and symptoms.

## Results

### Demographic and clinical data in schizophrenia patients and healthy controls

Table 1 showed no significant difference between schizophrenia patients and healthy controls in age, gender and education. There were significant gender differences in the age at onset of illness and duration of illness (p < 0.001 or p < 0.05) in schizophrenia patients (Table 2). In addition, there were no significant differences between male and female schizophrenia patients in any of the PANSS scores, the ratio of typical to atypical antipsychotics, and the antipsychotic dose (equivalent to chlorpromazine) (Table 2).

**Table 1:** Demographic characteristics in schizophrenia patients and healthy controls

Variables	Schizophrenia Patients (n = 192)	Healthy Controls (n = 160)	F or χ2	*P*-value
Gender (male/female)	112/80	80/80	2.44	> 0.05
Age (years)	47.3 ± 7.9	44.7 ± 11.0	3.59	> 0.05
Education (years)	10.8 ± 2.1	10.0 ± 3.6	3.81	> 0.05

Mean ± SD (standard deviation).

**Table 2:** Characteristics of patients with schizophrenia grouped by gender

	Male	Female	F or χ^2^	p-value
Age (years)	47.6 ± 8.5	47.6 ± 7.1	0.48	0.49
Education (years)	10.6 ± 2.1	11.2 ± 2.1	3.73	0.06
Age at onset of illness	22.5 ± 5.4	25.7 ± 7.3	11.13	< 0.01
Duration of illness	24.9 ± 9.3	21.3 ± 10.2	5.75	< 0.05
Antipsychotic type (typicals/atypicals)	13/99	9/71	0.00	0.83
Antipsychotic dose (CPZ equivalents)(mg)	365.9 ± 312.8	392.1 ± 218.9	0.35	0.56
Score on positive symptom scale	12.0 ± 4.5	13.5 ± 5.7	3.66	0.06
Score on negative symptom scale	17.9 ± 5.2	17.8 ± 5.8	0.03	0.87
Score on general psychopathology scale	27.4 ± 5.4	28.1 ± 6.2	0.63	0.43
Total PANSS score	61.1 ± 11.2	63.0 ± 14.4	0.94	0.33

Note: Mean ± SD. CPZ = chlorpromazine; PANSS = the positive and negative syndrome scale.

### Cognitive performance in schizophrenia patients and healthy controls

After controlling for age and education, multivariate analysis of covariance revealed significant differences in all cognitive domains between schizophrenia patients and healthy controls (F_1,191_ = 28.6, p < 0.001). Furthermore, diagnosis (schizophrenia patients vs healthy subjects) was significantly different for the MCCB total and all subtest scores (all p < 0.001). Male schizophrenia patients had significantly lower cognitive MCCB total and subtest scores (all p < 0.001) than male healthy subjects, with effect sizes ranging from 0.79 to 2.09. Female patients also had significantly lower cognitive MCCB total and subtest scores (all p < 0.001 or p < 0.01) than female healthy subjects, with effect sizes ranging from 0.42 to 1.35.

**Figure 1:**
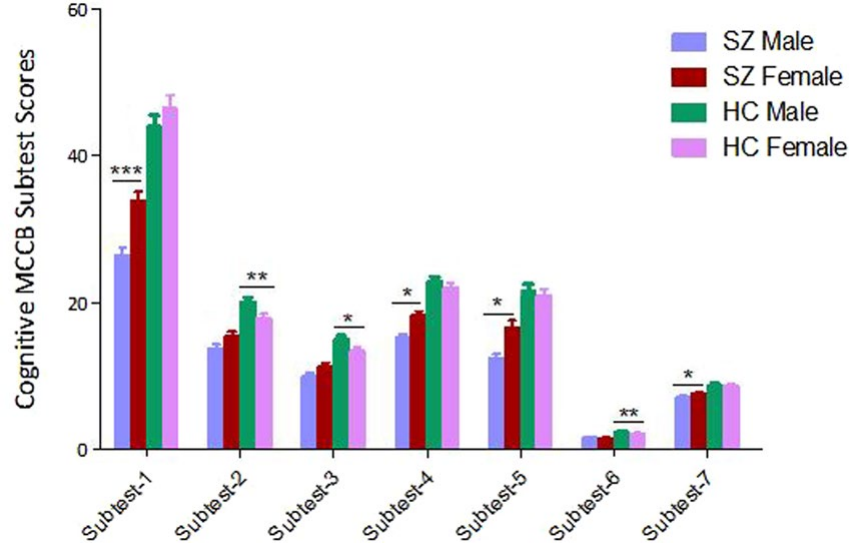
The cognitive test scores by MCCB in schizophrenia patients and healthy controls. Subtest-1: Symbol coding of processing speed; Subtest-2: Digital sequence of working memory; Subtest-3: Spatial span total of working memory; Subtest-4: Verbal learning; Subtest-5: Visual learning; Subtest-6: Reasoning and problem solving; Subtest-7: Social cognition. ***p < 0.001, **p < 0.01 and *p < 0.05. SZ: Schizophrenia patients; HC: Healthy controls. MCCB: MATRICS (Measurement and Treatment Research to Improve Cognition in Schizophrenia) Cognitive Consensus Batterys.

Multivariate analysis of covariance also revealed overall main effects for gender on symbol coding of processing speed, reasoning and problem solving (both p < 0.001). Also, analysis showed significant gender x diagnosis interaction effects on working memory, verbal learning and visual learning (p < 0.01 or p < 0.05). To further analyze these two-way interactions, we compared cognitive function in schizophrenia patients or healthy controls separately by gender. Male schizophrenia patients performed poorer than females on symbol coding of processing speed, verbal learning, visual learning, and social cognition (p < 0.001or p < 0.05), with effect sizes ranging from 0.44 to 0.69 (Fig. 1). Furthermore, the gender differences assessment on symbol coding of processing speed and visual learning passed Bonferroni corrections (all p < 0.01) but verbal learning and social cognition did not pass Bonferroni corrections (all p > 0.05). Male healthy controls had better cognitive performance than females in working memory (including digital sequence and spatial span total), reasoning and problem solving (p < 0.01 or p < 0.05), with effect sizes ranging from 0.39 to 0.42 (Fig. 1). Also, reasoning and problem solving and digital sequence of working memory passed Bonferroni corrections (all p < 0.05) but the spatial span total for working memory did not pass the Bonferroni corrections (p > 0.05) in healthy subjects.

### Relationship between cognitive performance and clinical variables in schizophrenia patients

Multivariate regression analysis showed that MCCB total score was independently associated with PANSS negative symptom score (beta = −0.497, t = −4.016, p < 0.001) and gender (beta = 3.237, t = 2.325, p < 0.05). These factors together predicted 12% of the variance of the total MCCB score.

The symbol coding score of processing speed was independently associated with age (beta = −0.556, t = −6.044, p < 0.001), gender (beta = 6.088, t = 4.346, p < 0.001), education (beta = 1.029, t = 2.974, p < 0.01), and PANSS negative symptom score (beta = −0.323, t = −2.631, p < 0.01). These factors together predicted 37% of the variance of the symbol coding score.

Verbal learning was independently associated with age (beta = −0.556, t = −6.044, p < 0.001) and gender (beta = 6.088, t = 4.346, p < 0.001). These factors together predicted 18% of the variance in verbal learning.

Visual learning was independently associated with the following variables: duration of illness (beta = −0.294, t = −5.744, p < 0.001), gender (beta = 2.844, t = 2.757, p < 0.01), and PANSS negative symptom score (beta = −0.220, t = −2.420, p < 0.05). These factors together predicted 25% of the variance in visual learning.

Social cognition was independently associated with education (beta = 0.191, t = 3.325, p < 0.01), and PANSS negative symptom score (beta = −0.053, t = −2.396, p < 0.001). These factors together predicted 9% of the variance in social cognition.

## Discussion

Compared to our previous study using RBANS cognitive measurement^9^, this study found more cognitive impairments on reasoning and problem solving, social cognition, processing speed and working memory in male than female schizophrenia patients. This study also extended our previous study of cognitive deficits in both male and female schizophrenia patients. Moreover, we found that male schizophrenia patients showed more severe cognitive deficits than female patients in verbal learning and visual learning that was consistent with our previous using RBANS study^9^. Furthermore, schizophrenia patients showed greater cognitive deficits in all examined cognitive domains than healthy controls for both genders, which were also consistent with our previous study using RBANS^9^.

A direct comparison of reasoning and problem solving between male and female patients showed no difference, which differed from normal subjects that males perform better in reasoning and problem solving than females. Therefore, our study suggest that male schizophrenia patients may have more cognitive impairment than females on reasoning and problem solving, which is consistent with previous finding^22^. Male healthy controls exemplified better reasoning and problem solving than females, which is also consistent with previous studies that showed cognitive advantages in males than females on spatial reasoning tasks^23^.

Furthermore, our study suggested that social cognitive deficits were worse in male than female schizophrenia patients, which was similar to previous observations^24^. Our results were in a line with some previous rodent studies mimicking schizophrenia-like behavior. For example, a reduction in social interaction was observed in male, but not in female rats following neonatal treatment with domoic acid^25^. Folate-deficient mice showed reduced social interaction in males but not females^26^. However, the pathophysiological mechanisms underlying the gender differences in the social cognition for schizophrenia patients were not clear. Rodent study showed that serotonin 5-HT2A receptor expression was reduced in the prefrontal cortex, hypothalamus, and midbrain of social isolated male mice^27^. Human study showed that 5-HT2A receptor −1438 A/G polymorphism in the promoter region may influence social cognitive function in patients with schizophrenia^28^. These results suggested that 5-HT2A receptor or their genetic variants may be contributed to the gender differences of social cognition in schizophrenia patients.

Processing speed is an important behavioural marker of the pathophysiology of schizophrenia^29^. This study found that male schizophrenia patients had worse symbol coding processing speed than females, which was consistent with previous studies^30,31^. Also, this study showed that gender was independently associated with the symbol coding processing speed in schizophrenia patients. In addition, the rat model also supported that males had worse processing speed that females^32^. These results suggested that gender difference exists in the processing speed in schizophrenia patients. The symbol coding processing speed in MCCB is similar with coding tasks of attention in RBANS (Table 3). However, our previous study did not find gender difference in attention for schizophrenia patients using RBANS^9^. The cognitive domains on attention did not distinguish subgroups (digit span and coding tasks) using our previous RBANS study, which may be the possible reason for the negative discovery of gender difference on attention^9^ (Table 3). Therefore, this distinction of subgroups using RBANS in cognitive analysis may be important in future studies.

**Table 3:** The lists of cognitive domains in MCCB and RBANS

MCCB	RBANS
**Processing speed**	**Immediate memory**
*Category fluency*	*List learning* ^*d*^
*Symbol coding* ^*a*^	*Story memory*
*Trail Making A*	**Visuospatial/constructional**
**Attention/Vigilance**	*Figure copy* ^*c*^
*CPT-IP*	*Line orientation*
**Working memory**	**Language**
*Digital sequence* ^*b*^	*Picture naming*
*Spatial span total* ^*c*^	*Semantic fluency tasks*
**Verbal learning**	**Attention**
*HVLT-R total* ^*d*^	*Digit span* ^*b*^
**Visual learning**	*Coding Tasks* ^*a*^
*BVMT-R total* ^*e*^	**Delayed memory**
**Reasoning and problem solving**	*List recall*
*Mazes* (*NAB*) total	*List recognition*
**Social cognition**	*Story recall*
*MSCEIT*	*Figure recall* ^*e*^

MCCB: Measurement and Treatment Research to Improve Cognition in Schizophrenia (MATRICS) Consensus Cognitive Battery; CPT-IP: Continuous Performance Test-Identical Pairs; HVLT: Hopkins Verbal Learning Test; BVMT: Brief Visuospatial Memory Test; NAB: Neuropsychological Assessment Battery; MSCEIT: Mayer-Salovey-Caruso Emotional Intelligence Test. RBANS: Repeatable Battery for the Assessment of Neuropsychological Status. ^a–e^Symbols indicate the positive findings in MCCB, which they have the similar cognitive measure parameters with RBANS.

A direct comparison of working memory between male and female patients showed no difference, which differed from normal subjects that that males perform better in working memory than females. Therefore, our study suggested that male schizophrenia patients may have more cognitive impairment than females, which is similar finding as previous report^33^. The rodent study also showed some less impairments in female than male in working memory^34,35^. The digital sequence of working memory in MCCB is equivalent to the digit span of attention in RBANS (Table 3). The spatial span total of working memory in MCCB has considerable overlap with the figure copy of visuospatial/constructional in RBANS (Table 3). Further, our previous study by Han *et al*. only addressed cognitive analysis by immediate memory, visuospatial/constructional, language and delayed memory and did not distinguish subgroups of these cognitive domains in RBANS^9^ (The details for subgroups of RBANS please see the Table 3). This could have led to some negative findings in gender differences of cognition on attention and visuospatial/constructional domains. In addition, other clinical study had opposite findings on gender difference in working memory impairment in schizophrenia patients which was that female schizophrenia patients had worse working memory than male patients^36^. Currently, we do not have a clearly explanation for the inconsistent findings. It could be due to inconsistent and complex factors tested that may result in heterogeneity in the schizophrenia diagnosis, the course of the disease, antipsychotic treatments, and ethnic background. Prefrontal cortex plays an important role in working memory^37^, which could warrant an imaging study to compare prefrontal cortical differences between male and female schizophrenia patients.

The impairments of verbal and visual learning were found in schizophrenia patients compared to healthy controls. Also, male schizophrenia patients had worse cognitive deficits than female patients on verbal and visual learning domains. Post-hoc comparisons of schizophrenia patients and healthy controls yield significant interactions between gender and diagnosis (schizophrenia patients vs. healthy controls). Verbal and visual learning were independently associated with gender in schizophrenia patients. These results supported that male schizophrenia patients had worse cognitive impairment than female patients on verbal and visual learning, which were consistent with other studies^36,38^. Verbal learning is related to immediate memory in RBANS (Table 3). Visual learning is related to figure recall of delayed memory in RBANS (Table 3). Therefore, male schizophrenia patients had significantly worse cognitive deficits than females on verbal and visual learning in the study, which were consistent with our previous RBANS results that male schizophrenia patients had worse immediate and delayed memory impairment than females^9^.

Compared to healthy controls, this study found significant cognitive deficits in all seven examined cognitive domains of MCCB in schizophrenia patients, which is consistent with ours and other previous studies^9,39–41^. Interestingly, our previous study observed a cognitive impairment trend in visuospatial/constructional domain in schizophrenia patients than healthy controls (p = 0.059)^9^. As described above, visuospatial/constructional domain mainly composed of figure copy and line orientation tasks in RBANS (Table 3). The figure copy of visuospatial/constructional is mainly overlapping with spatial span total of working memory The line orientation task of visuospatial/constructional is also mainly overlapping with trail making A for speed of processing in MCCB. However, in this study, the trail making A for speed of processing and spatial span total of working memory were found to incur significant impairment in schizophrenia patients compared to healthy controls. These results prompted that it is important to analyse the visuospatial/constructional cognitive domain by subgroup (figure copy and line orientation tasks) in using RBANS.

The effect of worse cognition for male schizophrenia patients compared to females in several cognitive domains has been consistently identified by both our MCCB and RBANS studies. However, the pathophysiological mechanism of gender difference of cognitive deficits in schizophrenia is not clear. Currently, neuroprotective effects of sex hormones (especially estrogens) are among the most studied and accepted explanation for gender difference in cognition^9,14,42,43^. Moreover, gender differences in response to antipsychotics treatment could also contribute to the differential cognitive impairments in schizophrenia patients^9,44–46^, while some studies did not support such claim^47–49^. In addition, some studies suggested that an earlier onset of schizophrenia was associated with the seriousness of the disease, including cognitive deficits^50^. Therefore, it should be noted that male schizophrenia patients generally had an earlier age of onset for the illness and longer duration of illness than female patients in this study, which might be one of reason that male schizophrenia patients performed worse cognitive deficits than females. However, other studies have failed to find differences in the cognitive profiles of individuals with early or late-onset schizophrenia^51,52^. Also, the excitation/inhibition imbalance theory of biological psychiatry may explain the relative preferential effect of schizophrenic neuropathology on particular mental processes^53^. This theory supports that gender differences in different cognitive domain may have specific pathophysiology in schizophrenia patients. Also, a study suggested that gender specific differences in neurotransmitter levels in the medial prefrontal cortex and hippocampus may contribute to the gender differences of cognitive deficits in schizophrenia patients^54^. Nevertheless, the pathophysiological mechanism that resulted in better performance of cognition for females than males in schizophrenia patients still needs to further be investigated.

In addition, our regression analysis revealed significant correlation between the symbol coding of processing speed score and PANSS negative symptom score, visual learning and duration of illness. These results supported that cognitive deficits was significantly related to the severity of negative symptoms and duration of illness of schizophrenia.

This study has some limitations. Since this research is a phenomenological study, it does not explain the molecular mechanisms of the gender differences or the pathophysiology of cognition in schizophrenia. First, the schizophrenia patients in this study were of the chronic patients. Although there were no gender differences in antipsychotic dose (CPZ equivalents) and antipsychotic type (typical/atypical), the duration of illness in male patients were longer than female patients. We could not exclude that the gender effects of cognition in schizophrenia patients might be caused by disease duration and chronic drug treatment. Second, the cross-sectional study provided a snapshot of the entire course of the disease. A future longitudinal study might help to reveal the effects of disease progression. Third, the patients were all inpatients and therefore these results might not be generalizable to all schizophrenia patients. Fourth, it should be mentioned that our results for spatial span of working memory, verbal learning and social cognition did not pass the Bonferroni corrections test. Therefore, these results should be carefully regarded. Furthermore, repeat analysis and larger sample size study might be conducted. Fifth, mental status, personal and family history of mental illness for healthy controls were assessed using unstructured interviews and were not assessed using structured interviews such as SCID. Therefore, we cannot completely exclude the healthy controls from the potential of being subject to weak psychiatric disorders.

In summary, compared to our previous using RBANS study, we have added new findings that male schizophrenia patients had worse cognitive impairment than females in symbol coding processing speed, working memory, reasoning and problem solving, and social cognition measured by MCCB. We also found significant gender differences in cognitive deficits in verbal and visual learning in schizophrenia patients, which were consistent with our previous study measured by RBANS. MCCB covers an equivalent range of cognitive measurements to RBANS and has additional advantaging in detecting deficits in reasoning, problem solving and social cognition in schizophrenia patients.

## Materials and Methods

### Ethics statement

A complete description of the study was given to all participants. All participants were provided with written informed consent. A psychiatrist evaluated all the participants to test whether they had the capacity to consent. The research procedure was explained during a detailed interview and the participants acknowledged they understood what they were required to do. Language appropriate to the participant’s level of comprehension and emotional readiness was used to maximise the understanding of the participants. If he/she was willing to participate in the research but was unable to understand the complexity of the research processes, the research was simultaneously described to the parent or guardian and the patient. The parent or guardian then explained the research process to the participants using those methods that gauged the participant’s interest and maximised their understanding. In these situations, written consent was provided by the parent or guardian on behalf of the participant. If the participant did not agree to participate in the research study, they were not discriminated against and were given the same treatment as the study participants.

### Consent from study population

Informed consent has been obtained from the patients or their parents and guardians to use the results for analysis and use information for online open-access publication before subjects were recruited. The study protocol was approved by the Institutional Review Board, Beijing HuiLongGuan Hospital, and all methods were performed in accordance with the relevant guidelines and regulations.

### Participants

One hundred and ninety two schizophrenia patients (male/female = 112/80) were recruited using a cross-sectional naturalistic design at Beijing HuiLongGuan Hospital, a Beijing City owned psychiatric hospital. The diagnoses for each patient were made by two independent and experienced psychiatrists and confirmed by the Chinese version of Structured Clinical Interview for *DSM-IV-TR Axis I Disorders. Research Version, Patient Edition (SCID-I/P)* ^55^. All patients were aged between 20 and 60 years, diagnosed with schizophrenia for at least 5 years, and were on stable doses of oral antipsychotic drugs for at least 12 months (ranging from 1 to 13 years) prior to entry into the study. Antipsychotic drugs consisted of monotherapy with atypical antipsychotics including clozapine 50–600 mg/day (n = 86), risperidone 2–10 mg/day (n = 52), quetiapine 300–1000 mg/day (n = 13), olanzapine 5–20 mg/day (n = 5), aripiprazole 10 mg/day (n = 1), and typical antipsychotics including haloperidol 12–50 mg/day (n = 5), chlorpromazine 200 mg/day (n = 1), perphenazine 14–30 mg/day (n = 7), sulpiride 100–1000 mg/day (n = 6), and others (n = 16). The mean antipsychotic dose (as chlorpromazine equivalents) was 379.2 ± 277.6 mg/day. Since admission, all patients received dietetically balanced hospital meals, which were occasionally supplemented by gifts (usually fruit), and patients had the opportunity to undertake about an hour of physical exercise every day.

### Clinical assessment

We obtained a complete medical history and physical examination from all participants, and participants with serious medical abnormalities were excluded. All schizophrenia patients were inpatients. All participants were Han Chinese recruited at the same period from the Beijing area. Both the schizophrenia patients and the healthy controls revealed a similar socioeconomic status and dietary patterns. Healthy controls (male/female = 80/80) were recruited from our local community. Current mental status and personal or family history of mental illness was assessed using unstructured interviews. None of the healthy controls presented a personal or family history of psychiatric disorders. Neither the patients with schizophrenia nor the healthy controls had a history of drug abuse or dependence.

Clinical assessments of patients were carried out by two psychiatrists who had more than five years of clinical practice experience and who were blind to the clinical status and treatment conditions assessed the participants’ psychopathology using the positive and negative syndrome scale (PANSS)^56^. To ensure consistent and reliable ratings, the two psychiatrists simultaneously attended a training session for standardizing their use of PANSS prior to the start of the study. Thereafter, they maintained an intra-class correlation coefficient of greater than 0.8 on the PANSS at repeated assessments during the course of this study.

### Cognitive assessment

All subjects were given cognitive function assessment using the MCCB^18,57^. A previous study by Zhou *et al*. at Beijing Huilong Guang Hospital showed that MCCB was valuable and reliable in assessing cognitive function in Chinese Han schizophrenia patients and health controls^19^. The MCCB is comprised of 10 standardized measures that are used to calculate cognitive functions in 7 domains and a global composite score. The MCCB battery consists of Trail Making Test Part A; Brief Assessment of Cognition in Schizophrenia: Symbol coding; Hopkins Verbal Learning Test (HVLT); Wechsler Memory Scale Spatial span; Digital Sequence Test; Neuropsychological Assessment Battery (NAB): Mazes; Brief Visuospatial Memory Test (BVMT); Category Fluency; Mayer-Salovey-Caruso Emotional Intelligence Test (MSCEIT): Managing Emotions; and the Continuous Performance Test: Identical Pairs. The seven MCCB domains were: processing speed, attention/vigilance, working memory, verbal learning, visual learning, reasoning and problem solving and social cognition^58^.

### Statistical Analysis

Group comparisons on the demographic and clinical variables used Chi squared or Fisher exact tests for the categorical variables and Student t-tests or analysis of variance (ANOVA) for the continuous variables. For the MCCB comparisons, we included age and education as covariates in the multivariate analyses of covariance (MANCOVA). We tested the main effect of diagnose (schizophrenia patients vs healthy controls), gender (male vs female) and diagnosis x gender. Effect sizes were also calculated for the two-way comparisons and represented the mean difference, in standard deviation units, between the groups of interest. MANCOVA was used to further analyse these differences in cognitive functions. Bonferroni corrections were applied to each test to adjust for multiple testing. To analysis the amount of variance in cognitive functioning explained by the psychopathological variables, first-order multiple linear models were estimated by using the least-squares estimator, while controlling for several potential confounders including age, gender, education, duration of illness and PANSS (positive, negative, general pathological symptoms and the total score). To select a subset of variables which were associated with the response, stepwise selection was used, where inclusion factors for variable selection were set at a p-value < 0.05.
